# Exploring potential causal associations between autoimmune diseases and colorectal cancer using bidirectional Mendelian randomization

**DOI:** 10.1038/s41598-024-51903-0

**Published:** 2024-01-18

**Authors:** Lu Chen, Feifan Wang, Hua Zhang, Baoshan Cao

**Affiliations:** 1 Department of Medical Oncology and Radiation Sickness, Peking University Third Hospital, Beijing, 100191 China; 2 Gastrointestinal Disease Diagnosis and Treatment Center, The First Hospital of Hebei Medical University, Shijiazhuang, 050000 China; 3 Research Center of Clinical Epidemiology, Peking University Third Hospital, Beijing, 100191 China

**Keywords:** Cancer, Gastrointestinal cancer, Genetic association study

## Abstract

Several observational studies have revealed an association between autoimmune diseases (AIDs) and colorectal cancer (CRC), although their causal association remained controversial. Therefore, our study used a two-sample Mendelian randomization (MR) analysis to verify the causal association between AIDs and CRC. We employed three common MR approaches, including inverse variance weighted (IVW), weighted median, and MR-Egger methods, to assess the causal association between type 1 diabetes (T1D), systemic lupus erythematosus, rheumatoid arthritis, psoriasis, multiple sclerosis, juvenile idiopathic arthritis, celiac disease, and primary sclerosing cholangitis (PSC) and CRC. The reverse MR analysis was performed to assess the possibility of reverse causation. To evaluate the validity of the analysis, we also performed sensitivity analysis, such as the heterogeneity test, the horizontal pleiotropy test, and the leave-one-out sensitivity analysis, and validated the results in the validation cohort. Our results showed that genetically predicted T1D was nominally associated with a lower risk of CRC (IVW OR = 0.965, 95% CI = 0.939–0.992, *P* = 0.012). However, genetic susceptibility to psoriasis nominally increased the risk of CRC (IVW OR = 1.026, 95% CI = 1.002–1.050, *P* = 0.037). Genetically predicted PSC had a significant causal effect on the increasing risk of CRC (IVW OR = 1.038, 95% CI = 1.016–1.060, *P* = 5.85 × 10^−4^). Furthermore, the MR analysis between PSC and the CRC validation cohort indicated consistent results. We found no causal association between genetically predicted other five AIDs and CRC (*P* > 0.05). The results of reverse MR analysis showed that genetically predicted CRC had no causal effect on T1D, psoriasis, and PSC (*P* > 0.05). The sensitivity analysis demonstrated that the results of the MR analysis were reliable. Our findings help to understand the causal association between AIDs and CRC, which deserves further investigation.

## Introduction

Colorectal cancer (CRC) is the third most common cancer and the second leading cause of cancer-related deaths globally^1^. It is estimated that by 2035, there could be up to 2.5 million new cases of colorectal cancer globally due to the acceleration of population aging^2^. Therefore, to improve CRC prevention and treatment, it is crucial to explore the risk factors and etiology of CRC^3^. The etiology of CRC is complicated and includes the accumulation of epigenetic and genetic risk factors, obesity, dietary habits, a sedentary lifestyle with little or no physical activity, alterations in gut microbiota, and others^4^. An increasing number of studies indicate that the immune system is critical to the occurrence, progression, and treatment of CRC^5^.

Autoimmune diseases (AIDs) are a group of complicated, chronic illnesses with unidentified causes. It is now generally accepted that AIDs are caused by excessive host immunoreaction due to impaired host immunoregulation^6^. AIDs can be systemic or can affect specific organs or body systems and affect approximately 10% of the world’s population^7^. Furthermore, AIDs could result in severe complications in multiple systems, which affects the population’s life expectancy. Some studies indicate that the global incidence of autoimmune diseases is increasing yearly^8,9^. Therefore, we need to pay more attention to these diseases.

As we all know, the dysfunction of the immune system may lead to the occurrence of AIDs. Besides the protection against pathogens, the immune system is also intensely involved in CRC prevention, development, and defense. Therefore, the immune system plays a crucial role in both AIDs and CRC, which might indicate a specific association between these two diseases. For example, numerous studies demonstrated the significance of the P2X7 receptor (P2X7R) in the pathogenesis of AIDs, such as systemic lupus erythematosus (SLE), rheumatoid arthritis (RA), multiple sclerosis (MS), and inflammatory bowel diseases (IBD)^10^. Furthermore, researchers have demonstrated that P2X7R promotes the proliferation and metastasis of CRC cells, and its expression level is negatively related to the overall survival of CRC patients^11^. Therefore, AIDs and CRC might have a shared genetic susceptibility. Many studies have supported the intimate relationship between AIDs and CRC, but they remain partially controversial. Certainly, IBD, such as Crohn’s disease and ulcerative colitis, increase the probability to develop CRC, which^.^is because the gut chronic inflammatory state inherent in patients with IBD increases the risk of CRC^12,13^. Moreover, IBD have been demonstrated as an essential risk factor for the development of CRC^14^. However, when it comes to the association between other AIDs and the risk of CRC, there are inconsistent findings in patients with type 1 diabetes (T1D), RA, primary sclerosing cholangitis (PSC), and psoriasis^15–17^. Current studies in this area are primarily observational, which might be susceptible to confounding factors such as disease development, treatment, and environmental exposure. Inevitably, observational studies might reverse causality and make it difficult to distinguish the actual causal association. For example, a previous observational study showed that patients with psoriasis had a higher risk of CRC than those without psoriasis^15^. However, a recent study indicated no significant association between psoriasis and CRC^18^.

Mendelian randomization (MR) is an analytical method used to evaluate actual causal relationships between diseases, especially when randomized controlled trials are not feasible to examine the causation and observational studies provide biased associations due to confounding or reverse causal relationships^19^. It uses single nucleotide polymorphisms (SNPs) as instrument variables (IVs) to deduce causal relationships. On the one hand, confounding factors can theoretically be avoided. On the other hand, genetic variation, which explains exposure, occurs before the outcome, thus excluding the interference of reverse causation^20,21^. However, when the precise biological function of a genetic variant used in MR analysis is unknown, traditional MR analysis is likely to provide erroneous conclusions about the direction of causation. Bidirectional MR can quantify the effect of each variable on causality and minimize the occurrence of such errors^22^.

In this study, we utilized summary data from large scale genome-wide association studies (GWAS) for the two-sample MR analysis to identify the causal effect of eight major AIDs, including T1D, SLE, RA, psoriasis, MS, juvenile idiopathic arthritis (JIA), celiac disease (CD), and PSC on the risk of CRC. Furthermore, we verified our results in the validation cohort. Finally, a reverse MR analysis was performed to assess the possibility of reverse causation.

## Materials and methods

### Study design

**Figure 1:**
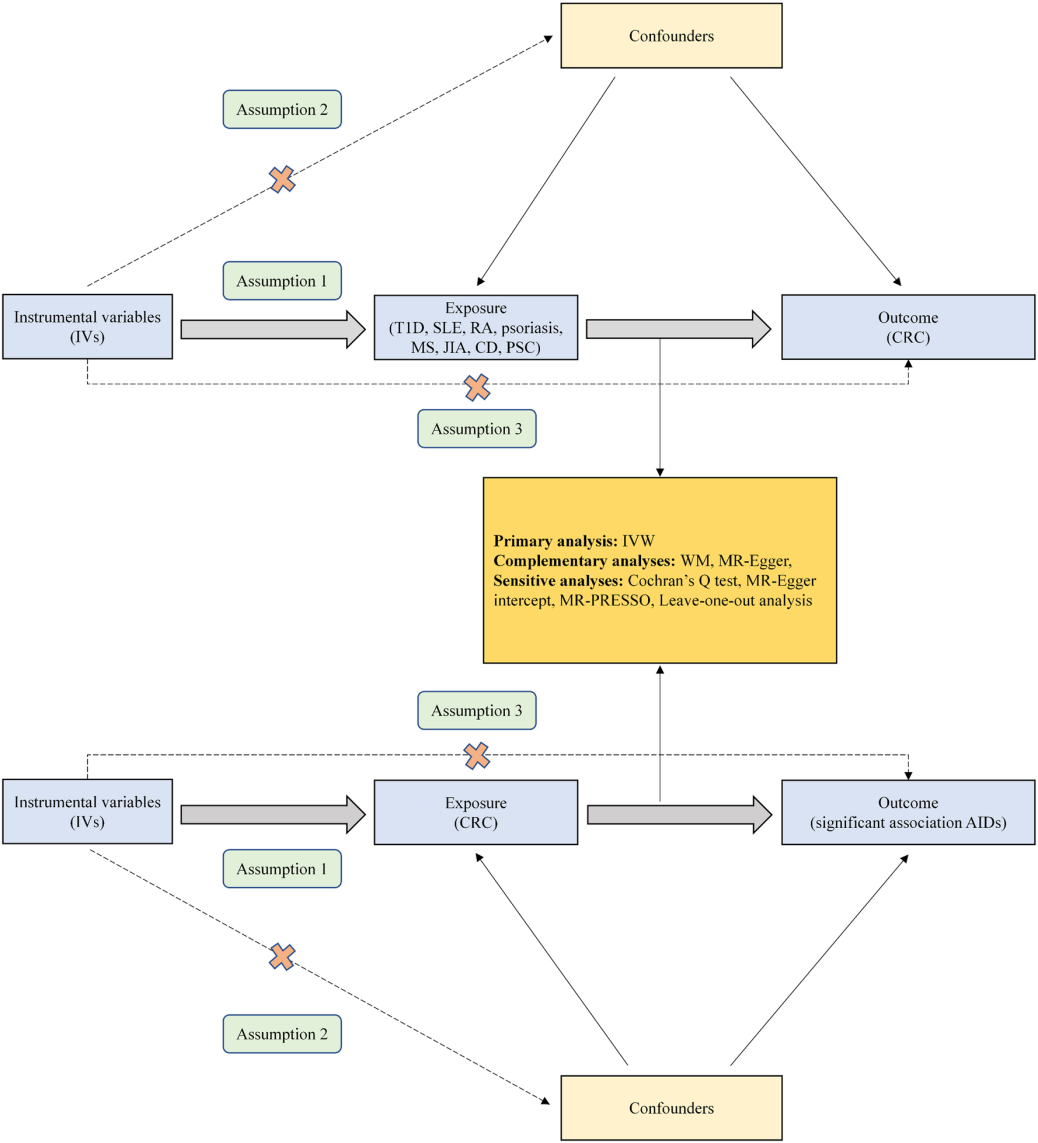
Flow diagram illustrating the core assumptions and study design of two-sample bidirectional MR analysis. Assumption 1: IVs are closely related to exposure. Assumption 2: IVs are not associated with confounders. Assumption 3: IVs influence the outcome only through the exposure. *T1D* type 1 diabetes, *SLE* systemic lupus erythematosus, *RA* rheumatoid arthritis, *MS* multiple sclerosis, *JIA* juvenile idiopathic arthritis, *CD* celiac disease, *PSC* primary sclerosing cholangitis, *CRC* colorectal cancer, *AIDs* autoimmune diseases.

Figure 1 shows the elementary study design of the two-sample MR analysis. In short, for the process of MR analysis, three assumptions must be satisfied: (1) relevance assumption: IVs are closely related to exposure; (2) independence assumption: IVs are not associated with confounders in the exposure-outcome association; (3) exclusivity assumption: IVs directly through exposure influence the outcome instead of through other pathways. Satisfying these assumptions, we are able to confirm that IVs directly influence the outcome through exposure instead of confounders, thus demonstrating the causal association between exposure and outcome. Moreover, we performed a reverse MR analysis to explore the effect of genetically predicted CRC on AIDs.

### Data sources

We obtained summary data from the genome-wide association study (GWAS) meta-analysis based on the European population for T1D (N = 520,580), SLE (N = 14,267), RA (N = 97,173), psoriasis (N = 44,161), MS (N = 115,803), JIA (N = 12,501), CD (N = 23,649), and PSC (N = 14,890)^23–30^. For CRC, we used two GWAS data, one from recently published GWAS data based on the European population (N = 185,616) as primary data for analysis, and the other from the UK Biobank study (N = 377,673) as validation cohort^31,32^. The detailed data sources were listed in Table S1.

### Instrumental variables selection

To satisfy the first assumption, we chose SNPs that were associated with exposure at a level of genome-wide significance (*P* < 5 × 10^−8^) and performed linkage disequilibrium (LD) clumping by setting r^2^ < 0.001 or clump distance > 10,000 kb to ensure the SNPs are independent. In addition, to determine whether there was a weak IV deviation, we calculated the *F*-statistics (*F* = R^2^ (n − k − 1)/k(1 − R^2^)), where R^2^ represents the variance of exposure explained by the IV, n is the sample size of the GWAS, and k is the total IVs. The *F*-statistics > 10 demonstrates a weak IV deviation. To eliminate the influence of confounders, we searched for pleiotropic SNPs of confounders in PhenoScanner (http://www.phenoscanner.medschl.cam.ac.uk/). After removing IVs that showed a strong association (*P* < 1E−5) with obesity, smoking, alcohol consumption, processed meat consumption, diabetes, inflammatory bowel disease, physical activity, leisure sedentary behaviors, cholesterol, and platelet, we utilized the remaining IVs for MR analysis^3,4,33,34^.

### Reverse Mendelian randomization analysis

We performed a reverse MR analysis using SNPs as IVs, which are associated with CRC, to demonstrate whether CRC has any causal effect on the identified significant association AIDs (i.e., CRC as exposure and the identified significant association AIDs as outcome). The IV selection conditions in reverse MR analysis were the same as in the primary MR analysis.

### Statistical analysis

MR analyses were conducted by the “TwoSampleMR” R package (version 0.5.6) and the “MRPRESSO” R package in R software (version 4.2.2). We used three common MR methods, including inverse variance weighted (IVW), weighted median (WM), and MR-Egger methods, to assess the causal association between eight major AIDs and CRC. For causal estimations of multiple SNPs, IVW is the main evaluation method for MR analysis because it has the most significant statistical power and can identify reliable causal estimates without directional pleiotropy^35^. So, we used IVW as the primary evaluation method. When heterogeneity exists in the analysis, random effects IVW can provide accurate causal estimation. We used the MR-Egger and the WM methods to supplement IVW estimates because they could provide reliable estimates in wider situations but were less efficient. The WM method provides consistent causal estimates when the valid IVs have more than 50% of the weight^36^. MR-Egger method can detect multiplicity by the intercept. However, its statistical power is impaired^37^.

Moreover, we carried out integrated sensitivity analyses to detect potential violations of the assumptions in the MR analysis. We used Cochran’s *Q* test to evaluate the heterogeneity across the individual causal effects (value of *P* < 0.05 suggesting heterogeneity). The MR-Pleiotropy Residual Sum and Outlier (MR-PRESSO) method was employed to explore outlier IVs and correct horizontal pleiotropy^38^. We used the MR-Egger intercept test to detect the horizontal and directional pleiotropy of IVs (value of *P* < 0.05 suggesting pleiotropy)^37^. Additionally, we conducted the Leave-one-out analysis to assess if a single SNP was responsible for the causal estimates or if it was the cause of bias.

Multiple comparisons were made by Bonferroni correction (*P* < 0.00625), which was regarded as evidence of statistical significance. However, *P* < 0.05 was assumed nominally significant of a causal association.

### Ethics approval

Ethical approval was waived because this study used the data from publicly available databases.

## Results

### Instrumental variables selection

First, we selected 81 IVs for T1D, 43 IVs for SLE, 76 IVs for RA, 55 IVs for psoriasis, 71 IVs for MS, 13 IVs for JIA, 37 IVs for CD, and 18 IVs for PSC, respectively. 2 SNPs of T1D, 2 SNPs of RA, 3 SNPs of psoriasis, 5 SNPs of MS, 3 SNPs of JIA, and 1 SNP of CD were excluded because they were not found in the dataset of the outcome. Next, we excluded 14SNPs of T1D, 1 SNP of SLE, 13 SNPs of RA, 6 SNPs of psoriasis, 9 SNPs of MS, 2 SNPs of JIA, 3 SNPs of CD, and 1 SNP of PSC because they were palindromic SNPs. Then, we searched for pleiotropic SNPs of confounders in PhenoScanner and removed them. Finally, we identified 42 SNPs for T1D, 34 SNPs for SLE, 46 SNPs for RA, 32 SNPs for psoriasis, 41 SNPs for MS, 7 SNPs for JIA, 17 SNPs for CD, and 11 SNPs for PSC to conduct MR analysis, respectively. The *F*-statistics of all IVs were > 10, indicating a slight possibility of a weak IV bias. The details of IVs for MR analysis are shown in Tables S2–S9.

### T1D and CRC

**Figure 2:**
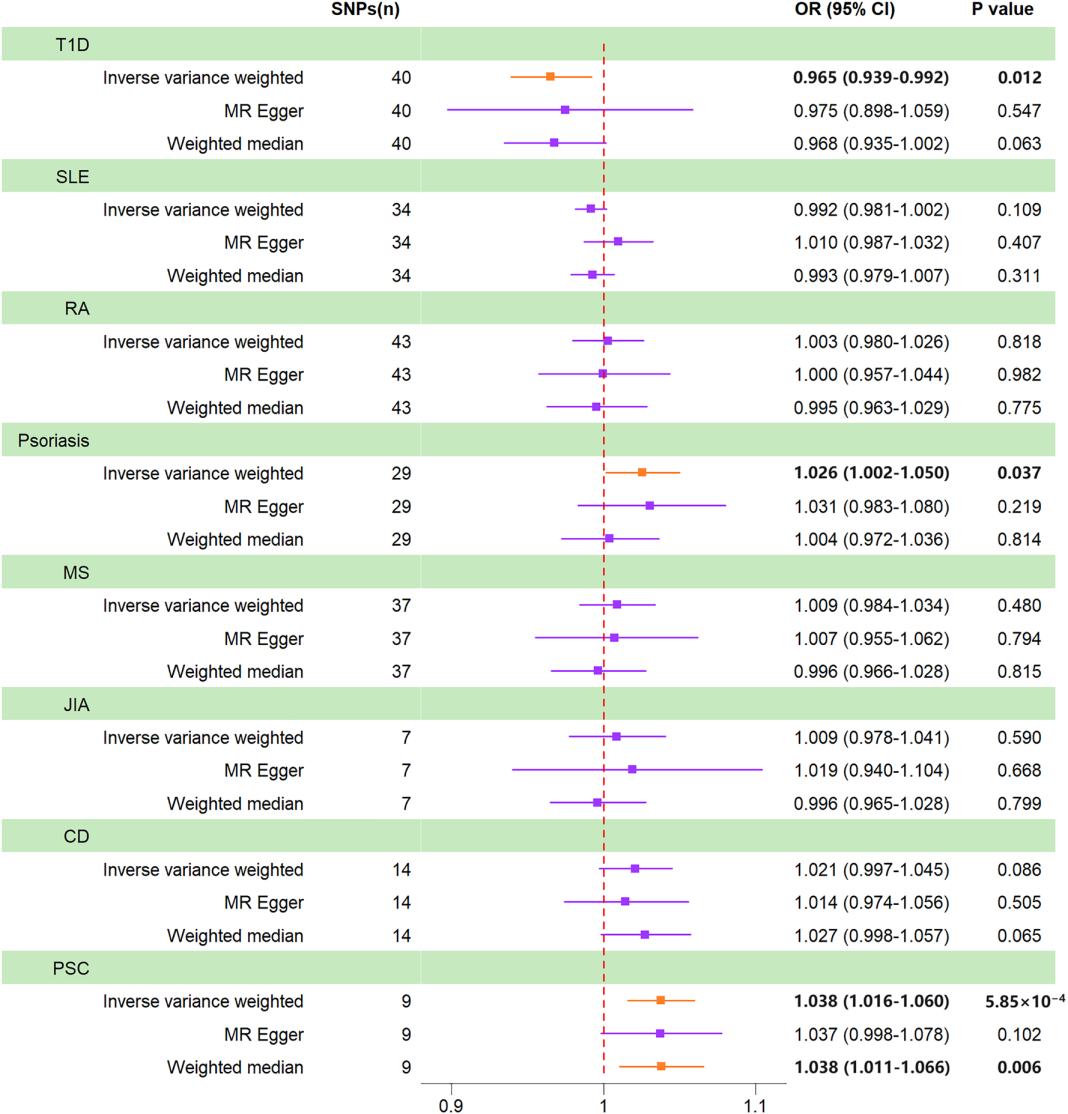
Forest plot for results of the Mendelian randomization analysis. *T1D* type 1 diabetes, *SLE* systemic lupus erythematosus, *RA* rheumatoid arthritis, *MS* multiple sclerosis, *JIA* juvenile idiopathic arthritis, *CD* celiac disease, *PSC* primary sclerosing cholangitis.

**Figure 3:**
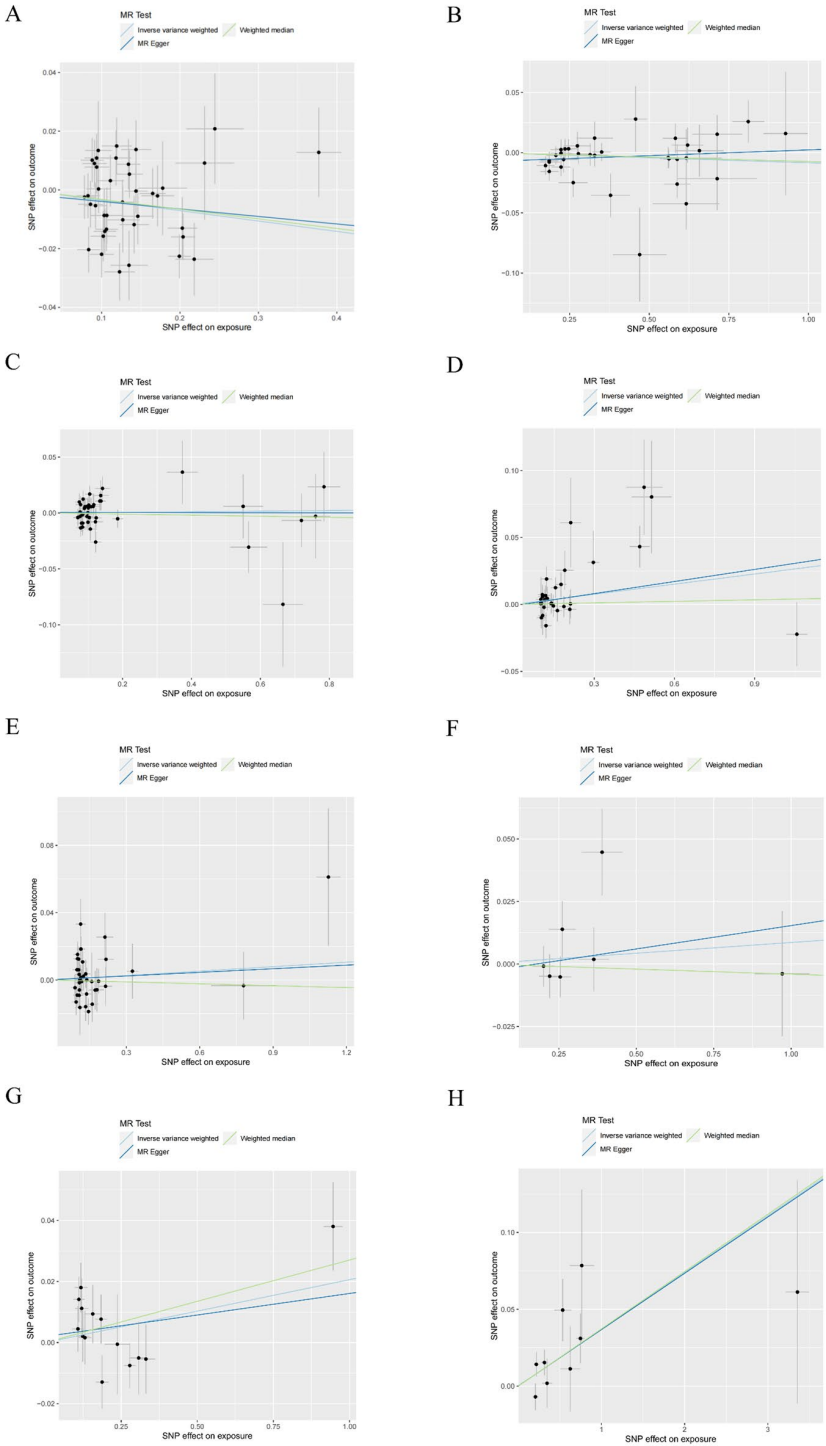
Scatter plots of the causal effect of T1D, SLE, RA, psoriasis, MS, JIA, CD, and PSC on CRC in the MR analysis. (**A**) T1D on CRC. The *F*-statistics of IVs were from 30.38 to 196.60. (**B**) SLE on CRC. The *F*-statistics of IVs were from 29.96 to 374.85. (**C**) RA on CRC. The *F*-statistics of IVs were from 29.70 to 321.46. (**D**) psoriasis on CRC. The *F*-statistics of IVs were from 30.60 to 728.17. (**E**) MS on CRC. The *F*-statistics of IVs were from 26.86 to 525.02. (**F**) JIA on CRC. The *F*-statistics of IVs were from 33.50 to 121.17. (**G**) CD on CRC. The *F*-statistics of IVs were from 29.83 to 920.79. (**H**) PSC on CRC. The *F*-statistics of IVs were from 27.32 to 574.41. *T1D* type 1 diabetes, *SLE* systemic lupus erythematosus, *RA* rheumatoid arthritis, *MS* multiple sclerosis, *JIA* juvenile idiopathic arthritis, *CD* celiac disease, *PSC* primary sclerosing cholangitis, *CRC* colorectal cancer.

The MR analysis results showed no causal association between T1D and CRC (IVW *P* = 0.080, MR-Egger *P* = 0.368, WM *P* = 0.070). However, Cochran’s *Q* test indicated significant heterogeneity (*P* < 0.001), and the MR-PRESSO global test showed horizontal pleiotropy (*P* < 0.001). After 2 outliers were removed (rs12742756 and rs2493411), we conducted MR analysis again. Based on Bonferroni correction, the result of the IVW method showed that genetically predicted T1D nominally decreased the risk of CRC (IVW OR = 0.965, 95% CI = 0.939–0.992, *P* = 0.012). In addition, the consistent impact direction was shown in other methods (Fig. 2). Although we excluded outliers detected in MR-PRESSO, there was still heterogeneity among SNPs (*P* = 0.007). We used the random effects IVW method to alleviate this problem. The MR-Egger intercept test showed no horizontal pleiotropy (*P* = 0.806) (Table 1). The scatter plot showed the nominally causal relationship between T1D and CRC using different MR methods (Fig. 3). Figure S1 shows the forest and funnel plots of all SNPs in MR analysis. The plot of leave-one-out sensitivity analysis determined that no SNP affected the overall results in this analysis (Fig. S1).

**Table 1:** Pleiotropy and heterogeneity test of the MR analysis. *T1D* type 1 diabetes, *SLE* systemic lupus erythematosus, *RA* rheumatoid arthritis, *MS* multiple sclerosis, *JIA* juvenile idiopathic arthritis, *CD* celiac disease, *PSC* primary sclerosing cholangitis.

Exposure	Pleiotropy test			Heterogeneity test		
	MR-Egger			Inverse variance weighted		
	Intercept	SE	*P*	Q	Q_df	Q_*p*val
T1D	− 0.001	0.006	0.806	62.724	39	0.007
SLE	− 0.007	0.004	0.087	38.249	33	0.243
RA	0.000	0.003	0.862	52.023	42	0.138
Psoriasis	− 0.001	0.004	0.814	38.203	28	0.095
MS	0.000	0.004	0.945	56.693	36	0.015
JIA	− 0.003	0.012	0.797	8.555	6	0.200
CD	0.002	0.005	0.698	18.577	13	0.137
PSC	0.000	0.008	0.986	8.261	8	0.408

### SLE and CRC

MR analysis showed no causal association between SLE and CRC (IVW *P* = 0.109, MR-Egger *P* = 0.407, WM *P* = 0.311) (Fig. 2). The Cochran’s *Q* test indicated no heterogeneity (*P* = 0.243), and the MR-Egger intercept test (*P* = 0.087) also showed no significant horizontal pleiotropy (Table 1). The MR-PRESSO detected no outlier (*P* = 0.254), which indicated our results were reliable. The scatter plot showed the causal relationship between SLE and CRC using different MR methods (Fig. 3). Figure S2 shows the forest and funnel plots of all SNPs in MR analysis. The plot of leave-one-out sensitivity analysis determined that no abnormal IV in our analysis affected the overall results (Fig. S2).

### RA and CRC

MR analysis showed no causal association between RA and CRC (IVW *P* = 0.900, MR-Egger *P* = 0.573, WM *P* = 0.743). The Cochran’s *Q* test showed significant heterogeneity (*P* < 0.001), and the MR-PRESSO global test showed horizontal pleiotropy (*P* < 0.001). After 3 outliers were removed (rs114435492, rs17534670, and rs3087243), the MR analysis was performed again. The results of MR analysis did not change (IVW *P* = 0.818, MR-Egger *P* = 0.982, WM *P* = 0.775) (Fig. 2). We found no heterogeneity in our MR analysis (*P* = 0.138), and the MR-Egger intercept test (*P* = 0.862) also showed no significant horizontal pleiotropy (Table 1). The scatter plot showed the causal relationship between RA and CRC using different MR methods (Fig. 3). The forest and funnel plots of all SNPs in MR analysis were displayed in Fig. S3. The plot of leave-one-out sensitivity analysis indicated that no IV influenced the overall results in this analysis (Fig. S3).

### Psoriasis and CRC

MR analysis showed no causal association between psoriasis and CRC (IVW *P* = 0.803, MR-Egger *P* = 0.692, WM *P* = 0.607). The Cochran’s *Q* test showed significant heterogeneity (*P* < 0.001), and the MR-PRESSO global test showed horizontal pleiotropy (*P* < 0.001). After 3 outliers were removed (rs10893885, rs582757, and rs9481169), the MR analysis was performed again. However, the results of MR analysis changed. After Bonferroni correction for multiple tests, we found that genetically predicted psoriasis nominally increased the risk of CRC (IVW OR = 1.026, 95% CI = 1.002–1.050, *P* = 0.037) (Fig. 2). The heterogeneity in our MR analysis was eliminated (*P* = 0.095), and the MR-Egger intercept test (*P* = 0.814) showed no horizontal pleiotropy (Table 1). The scatter plot showed the nominally causal relationship between psoriasis and CRC using different MR methods (Fig. 3). The forest and funnel plots of all SNPs in MR analysis were displayed in Fig. S4. The plot of leave-one-out sensitivity analysis determined that no SNP affected the overall results in this analysis (Fig. S4).

### MS and CRC

MR analysis showed no causal association between MS and CRC (IVW *P* = 0.341, MR-Egger *P* = 0.904, WM *P* = 0.821). The Cochran’s *Q* test showed significant heterogeneity (*P* < 0.001), and the MR-PRESSO global test showed horizontal pleiotropy (*P* < 0.001). After 4 outliers were removed (rs4325907, rs57116599, rs7855251, and rs12365699), the MR analysis was performed again. The results of MR analysis did not change (IVW *P* = 0.480, MR-Egger *P* = 0.794, WM *P* = 0.815) (Fig. 2). The Cochran’s *Q* test still showed heterogeneity (*P* = 0.015), and we used the random effects IVW method to alleviate this problem. The MR-Egger intercept test showed no horizontal pleiotropy (*P* = 0.945) (Table 1). The scatter plot showed the causal relationship between MS and CRC using different MR methods (Fig. 3). The forest and funnel plots of all SNPs in MR analysis were displayed in Fig. S5. The plot of leave-one-out sensitivity analysis indicated that no IV influenced the overall results in this analysis (Fig. S5).

### JIA and CRC

MR analysis showed no causal association between JIA and CRC (IVW *P* = 0.590, MR-Egger *P* = 0.668, WM *P* = 0.799) (Fig. 2). The Cochran’s *Q* test indicated no heterogeneity (*P* = 0.200), and the MR-Egger intercept test (*P* = 0.797) showed no significant horizontal pleiotropy (Table 1). The MR-PRESSO global test (*P* = 0.265) also showed a consistent result. The scatter plot showed the causal relationship between JIA and CRC using different MR methods (Fig. 3). Figure S6 shows the forest and funnel plots of all SNPs in MR analysis. The plot of leave-one-out sensitivity analysis demonstrated that no IV influenced the overall results in this analysis (Fig. S6).

### CD and CRC

MR analysis showed no causal association between CD and CRC (IVW OR = 1.016, 95% CI = 0.983–1.051, *P* = 0.343). However, Cochran’s *Q* test indicated significant heterogeneity (*P* < 0.001), and the MR-PRESSO global test showed significant horizontal pleiotropy (*P* < 0.001). After 3 outliers were removed (rs1018326, rs10790269, and rs79758729), we conducted MR analysis again. The results of MR analysis did not change, which indicated no causal association between CD and CRC (IVW *P* = 0.086, MR-Egger *P* = 0.505, WM *P* = 0.065) (Fig. 2). The heterogeneity in our MR analysis was eliminated (*P* = 0.137), and the MR-Egger intercept test (*P* = 0.698) showed no horizontal pleiotropy (Table 1). The scatter plot showed the causal relationship between CD and CRC using different MR methods (Fig. 3). The forest and funnel plots of all SNPs in MR analysis were displayed in Fig. S7. The plot of leave-one-out sensitivity analysis demonstrated that no abnormal IV influenced the results in MR analysis (Fig. S7).

### PSC and CRC

The result of the WM method showed that genetically predicted PSC increased the risk of CRC (WM OR = 1.033, 95% CI = 1.005–1.061, *P* = 0.019), but the IVW method showed that there was no causal association between PSC and CRC (*P* = 0.206). However, Cochran’s *Q* test indicated significant heterogeneity (*P* = 0.001), and the MR-PRESSO global test showed horizontal pleiotropy (*P* = 0.003). After 2 outliers were removed (rs41316239 and rs72837826), we conducted MR analysis again. After Bonferroni correction for multiple tests, PSC and CRC showed a significant causal association. We found that genetically predicted PSC could increase the risk of CRC using the IVW method (IVW OR = 1.038, 95% CI = 1.016–1.060, *P* = 5.85 × 10^−4^). Additionally, the consistent impact direction was shown in other methods (Fig. 2). The heterogeneity in our MR analysis was eliminated (*P* = 0.408), and the MR-Egger intercept test (*P* = 0.986) showed no horizontal pleiotropy (Table 1). The scatter plot showed the causal relationship between PSC and CRC using different MR methods (Fig. 3). The forest and funnel plots of all SNPs in MR analysis were displayed in Fig. S8. The plot of leave-one-out sensitivity analysis determined that no IV affected the overall results in MR analysis (Fig. S8). Furthermore, the same analysis was performed between PSC and the CRC validation cohort, and the results were consistent (IVW OR = 1.0005, 95% CI = 1.0001–1.0010, *P* = 0.025). The detailed results were showed in Table S10.

### Reverse MR analysis

Finally, we performed a reverse MR analysis between CRC and T1D, psoriasis, and PSC, but we did not find reverse causal relationships between them in all MR methods (Table 2). The details of IVs for CRC were shown in Table S11. The Cochran’s *Q* test showed partial heterogeneity in the reverse MR analysis, and the MR-Egger intercept test showed no horizontal pleiotropy in the reverse MR analysis (Table S12). The plots of other sensitivity analyses were showed in Figs. S9–S11.

**Table 2:** Reverse causal association between CRC and AIDs. *T1D* type 1 diabetes, *PSC* primary sclerosing cholangitis, *CRC* colorectal cancer, *MR* Mendelian randomization.

Exposure	Outcome	MR method	SNPs (n)	OR (95% CI)	*P*
CRC	T1D	Inverse variance weighted	104	1.046 (0.980–1.118)	0.178
		MR-Egger		0.966 (0.818–1.140)	0.682
		Weighted median		1.022 (0.946–1.105)	0.577
CRC	Psoriasis	Inverse variance weighted	104	0.989 (0.917–1.066)	0.773
		MR-Egger		1.013 (0.839–1.223)	0.891
		Weighted median		0.932 (0.843–1.031)	0.170
CRC	PSC	Inverse variance weighted	64	1.071 (0.929–1.233)	0.345
		MR-Egger		0.948 (0.675–1.331)	0.759
		Weighted median		1.027 (0.847–1.245)	0.786

## Discussion

We used the bidirectional two-sample MR analysis for the first time to systematically reveal the causal association between eight major AIDs and CRC. After Bonferroni correction for multiple tests, our study showed that genetically predicted T1D nominally decreased the risk of CRC (IVW OR = 0.965, 95% CI = 0.939–0.992, *P* = 0.012). However, genetically predicted psoriasis was nominally associated with an increased risk of CRC (IVW OR = 1.026, 95% CI = 1.002–1.050, *P* = 0.037). PSC and CRC showed a significant causal association. We found that genetically predicted PSC was significantly associated with an increased risk of CRC (IVW OR = 1.038, 95% CI = 1.016–1.060, *P* = 5.85 × 10^−4^). Furthermore, the MR analysis between PSC and the CRC validation cohort indicated consistent results (IVW OR = 1.0005, 95% CI = 1.0001–1.0010, *P* = 0.025). However, we found no causal association between five other AIDs (SLE, RA, MS, JIA, and CD) and CRC. The results of reverse MR analysis demonstrated that genetically predicted CRC had no causal effect on T1D, psoriasis, and PSC (*P* > 0.05). As far as we know, our study is the first to ascertain causal associations between AIDs and CRC using MR analysis.

Studies of the association of T1D with cancers were limited. In a retrospective study in Australia, T1D was positively correlated with CRC in females but found no association in males^39^. Conversely, a meta-analysis of observational studies showed no significant association between T1D and the risk of CRC (RR = 0.90, 95% CI = 0.61–1.31, *I*^2^ = 20.9)^16^. However, our study demonstrated that genetic vulnerabilities to T1D nominally decreased the risk of CRC using MR analysis. The discrepancies between our results and previous observational findings might be caused by various measurement errors, underlying biases, and confounding factors (e.g., use of insulin and incidence of complications) in the observational studies. These factors might reverse causality and make it challenging to clarify the real causal relationship. Based on previous studies, the nominal association between T1D and a decreased risk of CRC might involve the following aspects. First, mast cells have been discovered to have a protective anti-tumor role in the development of intestinal tumors^40^. There were very few mast cells in a healthy pancreas but a rise in pancreatic mast cell density appeared among individuals with T1D^41^. Second, T1D could result in severe complications in multiple systems, which might affect the population’s life expectancy. However, CRC is an age-related disease, and early mortality from T1D could reduce CRC prevalence. Third, T1D was genetically heterogeneous, and for the most common forms of T1D, alleles of human leukocyte antigen (HLA)-DR were an essential determinant of T1D^42^. Nevertheless, some research indicated that HLA-DR was a favorable prognostic indicator in CRC^43^. Forth, an MR study showed a positive correlation between the *Bifidobacterium* genus and the incidence of T1D^44^. On the other hand, an increased abundance of *Bifidobacterium* could enhance antitumor immunity and inhibit the development of CRC^45^.

Our study indicated that psoriasis nominally increased the risk of CRC, which was consistent with a previous meta-analysis of observational studies^15^. The possible mechanisms may be due to the following reasons. First, chemokine CCL27 played an essential role in psoriasis. Meanwhile, it was also involved in the cellular proliferation and migration of tumor cells^46^. Second, IL-17 and IL-22 are related to the etiology of autoimmune diseases such as psoriasis and numerous inflammatory-associated cancers, including CRC^47^. Third, Chang et al. found that several cancer metabolic pathways, including those of tryptophan metabolism and lipid biosynthesis proteins, were significantly enhanced in the blood microbiome in patients with psoriasis based on 16S rRNA metagenome sequences^48^. At the same time, tryptophan metabolism and lipid biosynthesis proteins were significantly associated with the development of CRC^49,50^.

Generally, PSC was significantly associated with susceptibility to colorectal cancer, consistent with a previous meta-analysis of observational studies^17^. The explanation for this phenomenon may involve several complicated factors. First, PSC was closely linked to IBD, and more than two-thirds of PSC patients were accompanied by IBD^51^. IBD has been demonstrated to be a risk factor for CRC. In addition, compared to patients with IBD alone, those with IBD and PSC had a fivefold increased risk of developing CRC^52^. These evidences indicated that PSC could directly increase the risk of CRC or indirectly promote CRC through IBD. Second, recent research found that the number of plasma cells that secreted immunoglobulin G and FOXP3^+^ CD4^+^ T cells that produced the cytokine IL-17 was increased in PSC patients’ right colonic tissue^53^. This indicated that the burden of intestinal inflammation was heavy in PSC patients, which might cause CRC. Third, Nakamoto et al. found *K. pneumoniae*, *Proteus mirabilis,* and *Enterococcus gallinarum* were prevalently detected in patients with PSC and demonstrated that PSC-derived *K. pneumoniae* could damage the intestinal epithelium in animal experiments^54^. The disturbance of intestinal flora could damage the intestinal barrier and result in bacterial translocation, which increased the risk of CRC. The recent discovery of alteration in intestinal fungi composition also reinforced the role of gut dysbiosis in PSC promoting CRC^55^. Fourth, Gao et al. used multidrug resistance protein 2 knockout (Mdr2^−/−^) mice to mirror human PSC and found that Mdr2^−/−^ mice appeared to have alterations in fecal bile acid composition and enhanced colitis susceptibility. In Mdr2^−/−^ mice, ursodeoxycholic acid treatment attenuated colitis susceptibility, which demonstrated that bile acid metabolism might be involved in promoting colitis and CRC by PSC^56^.

This study demonstrated no causal association between five other AIDs (SLE, RA, MS, JIA, and CD) and CRC, which was consistent with previous partial observational studies^57–59^. However, there were also partial studies inconsistent with our results. A Meta-analysis reported that patients with RA had a decreased risk of CRC^60^. A cohort study showed the risk of CRC was higher in MS patients than in healthy people^61^. Above all, the previously observed association between RA and MS and CRC could be mediated by hitherto unknown confounding factors. Therefore, further research is required to clarify the association between these AIDs and CRC using robust designs to eliminate confounding and survival bias. It is necessary to conduct cohort studies with large sample size adjusting for potential confounders. Current observational studies inevitably have confounding factors or reverse causation bias. Thus, animal experiments are equally necessary to eliminate these influences and clarify the underlying mechanisms between AIDs and CRC. In addition, we first reported that genetically predicted CRC had no effect on T1D, psoriasis, and PSC, which demonstrated the associations of these diseases were unidirectional.

Our study has several major strengths. First, the most primary strength was the MR design, which avoided confounding factors and reverse causality in previous observational studies. Second, we performed a variety of sensitivity analyses and explained the potential mechanisms involved in these associations combined with previous studies, which made our conclusions more convincing. Third, we demonstrated that T1D, psoriasis, and PSC had causal effects on CRC. Although the odds ratio was not high, these described associations might have clinical implications. For example, we suggested that it was necessary for us to strengthen the prevention and screening of CRC in patients with AIDs, especially those with the use of immunosuppressive treatments. Fourth, we indicated that there were causal associations between AIDs and CRC. The elucidation of the mechanisms between AIDs and CRC might allow us to exploit these mechanisms to monitor and treat these two diseases in the future.

There are several limitations to our study. First, we selected the IVs and GWAS data based on the European population to construct MR analysis in this study. It means whether our results are consistent in other ethnic populations remains to be verified. Second, although the GWAS data of AIDs and CRC came from different samples in our study, it is inevitable that there may be a sample overlap. However, sample overlap in the two-sample MR analysis may lead to an overestimation of the results. Third, we confirmed no causal association between five other AIDs (SLE, RA, MS, JIA, and CD) and CRC. However, we cannot exclude the possibility that this association was not detected due to insufficient sample size at present. With larger sample sizes of GWAS in the future, the association between AIDs and CRC might become apparent. Fourth, we could not rule out the possibility that the association between AIDs and CRC may be non-linear. Current MR methods assume that the exposure–outcome correlation is linear. Furthermore, the causal association and underlying mechanisms between AIDs and CRC must be further confirmed in animal experiments or cohort studies with large sample size.

In summary, our study demonstrated that genetic vulnerabilities to psoriasis and PSC could increase the risk of CRC using MR analysis. Genetically predicted T1D was associated with a decreased risk of CRC. However, there was no evidence to show a causal association between five other AIDs (SLE, RA, MS, JIA, and CD) and CRC. The results of reverse MR analysis demonstrated that genetically predicted CRC had no causal effect on T1D, psoriasis, and PSC. Our findings help to comprehend the causal association between AIDs and CRC, which deserves further investigation.

### Supplementary Information

Supplementary Information.

## Data Availability

The original contributions presented in the study are included in the article/Supplementary Material, further inquiries can be directed to the corresponding author.
