# Supplementary material for: Exploring potential causal associations between autoimmune diseases and colorectal cancer using bidirectional Mendelian randomization
Source: Sci Rep. 2024 Jan 18;14:1557. doi: 10.1038/s41598-024-51903-0 (PMC10796354; doi:10.1038/s41598-024-51903-0)
Supplement: Supplementary file 1 — Supplementary Information. [file 41598_2024_51903_MOESM1_ESM.pdf]

Exploring potential causal association between autoimmune diseases and colorectal cancer using bidirectional Mendelian randomization

Lu Chen[1,†], Feifan Wang [2,†], Hua Zhang[3], Baoshan Cao[1,*]

1 Department of Medical Oncology and Radiation Sickness, Peking University Third Hospital, Beijing 100191, China

2 Gastrointestinal Disease Diagnosis and Treatment Center, The First Hospital of Hebei Medical University, Shijiazhuang 050000, China

3 Research Center of Clinical Epidemiology, Peking University Third Hospital, Beijing 100191, China

* Correspondence: caobaoshan0711@aliyun.com

† These authors contributed equally to this work.

Acknowledgments: We are grateful to the IMSGC and related institutions for making the GWAS summary data publicly available, and grateful to all the researchers and participants who contributed to those studies.

Table S1. Details of the GWAS datasets included in the two-sample Mendelian randomization

Phenotype	Race	PMID	Participants
type 1 diabetes	European	34012112	18,942 cases and 501,683 controls
systemic lupus erythematosus	European	26502338	5,201 cases and 9,066 controls
rheumatoid arthritis	European	36333501	22,350 cases and 74,823 controls
psoriasis	European	34927100	15,967cases and 28,194 controls
multiple sclerosis	European	31604244	14,802cases and 26,703 controls
juvenile idiopathic arthritis	European	33106285	3,305 cases and 9,196 controls
celiac disease	European	22057235	11,812 cases and 11,837 controls
primary sclerosing cholangitis	European	27992413	2,871 cases and 12,019 controls
colorectal cancer	European	36539618	78,473 cases and 107,143 controls
colorectal cancer (validation)	European	UK Biobank	5,657 cases and 372,016 controls

Table S2. 42 SNPs used as instrumental variables for T1D in MR analysis

SNP	A1	A2	β	SE	*P*-value	*F*-statistics
rs10224046	G	T	0.0858	0.0154	2.71E-08	30.90
rs1050979	G	A	0.1062	0.0141	5.65E-14	56.49
rs10751776	C	A	0.0781	0.0141	2.67E-08	30.93
rs10801128	G	A	0.0961	0.0157	8.98E-10	37.54
rs11203203	A	G	0.1438	0.0144	1.81E-23	99.66
rs113374757	T	C	-0.1713	0.0208	1.63E-16	68.01
rs114278107	G	T	-0.1462	0.0199	1.85E-13	54.16
rs114378220	T	C	0.1779	0.0304	5.11E-09	34.15
rs12128789	C	T	0.1270	0.0215	3.73E-09	34.76
rs12257077	T	C	0.2312	0.0370	3.91E-10	39.16
rs12464462	G	A	-0.0880	0.0143	8.61E-10	37.62
rs12927355	T	C	-0.2039	0.0152	4.41E-41	180.20
rs13259300	C	A	-0.0922	0.0147	3.28E-10	39.50
rs1350275	G	T	-0.0937	0.0153	8.86E-10	37.56
rs1574285	T	G	-0.1265	0.0142	4.27E-19	79.75
rs17623914	C	T	-0.1349	0.0234	7.97E-09	33.28
rs1947178	G	A	-0.1033	0.0171	1.67E-09	36.32
rs202535	A	C	-0.1414	0.0185	1.79E-14	58.75
rs229527	A	C	0.1041	0.0141	1.82E-13	54.19
rs238265	G	T	-0.0908	0.0152	2.08E-09	35.90
rs2543537	T	C	-0.0834	0.0143	5.59E-09	33.97
rs2611211	T	C	-0.1439	0.0187	1.39E-14	59.25
rs3087243	A	G	-0.1991	0.0142	1.16E-44	196.60
rs34593439	A	G	-0.2181	0.0241	1.54E-19	81.75
rs35327136	A	C	-0.1192	0.0190	3.37E-10	39.45
rs3802214	C	T	-0.1066	0.0192	2.96E-08	30.73
rs4548024	C	T	-0.0957	0.0167	9.95E-09	32.85
rs56994090	C	T	-0.1343	0.0146	3.60E-20	84.63
rs61759532	T	C	0.1184	0.0186	1.91E-10	40.56

rs663743	A	G	-0.1000	0.0151	3.50E-11	43.87
rs6908626	T	G	0.2029	0.0185	6.14E-28	120.06
rs7068821	T	G	-0.1651	0.0163	5.07E-24	102.18
rs722988	C	T	0.0826	0.0144	9.78E-09	32.89
rs72838204	T	C	0.3769	0.0281	4.26E-41	180.25
rs7668577	C	A	0.0937	0.0152	7.26E-10	37.95
rs7776597	G	A	0.2444	0.0364	1.82E-11	45.16
rs7795896	T	C	-0.1354	0.0164	1.58E-16	68.07
rs855330	C	T	0.1112	0.0169	4.89E-11	43.22
rs9517712	C	T	-0.1021	0.0158	1.06E-10	41.70
rs6434435	A	G	-0.1229	0.0191	1.23E-10	41.42
rs12742756	G	A	-0.0831	0.0151	3.54E-08	30.38
rs2493411	C	T	0.1271	0.0223	1.28E-08	32.36

Table S3. 34 SNPs used as instrumental variables for SLE in MR analysis

SNP	A1	A2	β	SE	*P*-value	*F*-statistics
rs10048743	G	T	0.2311	0.0412	2.04E-08	31.46
rs1078324	A	C	-0.7133	0.0782	7.11E-20	83.28
rs10912578	A	G	0.2469	0.0310	1.65E-15	63.45
rs1143679	A	G	0.5822	0.0400	5.03E-48	212.00
rs12094036	C	T	-0.3285	0.0579	1.37E-08	32.24
rs13019891	T	G	-0.5621	0.0290	1.65E-83	374.85
rs13136219	T	C	-0.1744	0.0278	3.50E-10	39.37
rs13332649	G	A	-0.3147	0.0376	5.43E-17	70.17
rs143123127	A	G	0.4700	0.0840	2.23E-08	31.28
rs143810596	G	T	-0.6162	0.1126	4.41E-08	29.96
rs1464446	T	G	-0.3285	0.0401	2.79E-16	66.94
rs150180633	T	C	0.9282	0.0690	2.66E-41	181.19
rs2431697	C	T	-0.2231	0.0293	2.60E-14	58.01
rs2459611	C	T	-0.2614	0.0452	7.62E-09	33.37
rs2573219	C	A	0.5878	0.0429	1.13E-42	187.47
rs268124	C	T	-0.1863	0.0324	8.60E-09	33.13
rs34703115	C	T	-0.6162	0.1048	4.08E-09	34.58
rs35000415	T	C	0.5878	0.0415	1.86E-45	200.23
rs35251378	A	G	-0.2357	0.0324	3.61E-13	52.84
rs353608	A	G	-0.1863	0.0280	2.93E-11	44.22
rs4274624	C	T	0.5596	0.0327	9.73E-66	293.25
rs4388254	T	C	0.3784	0.0604	3.71E-10	39.26
rs4661543	T	G	-0.2744	0.0424	9.40E-11	41.94
rs4916215	C	T	-0.2231	0.0340	5.07E-11	43.15
rs58688157	G	A	-0.2231	0.0336	2.97E-11	44.20
rs58721818	T	C	0.6575	0.0756	3.38E-18	75.66
rs6889239	C	T	0.2776	0.0317	2.19E-18	76.51
rs7097397	A	G	-0.1863	0.0287	8.60E-11	42.12

rs73050535	T	C	-0.7133	0.1241	9.11E-09	33.02
rs7768653	C	T	0.2070	0.0297	3.11E-12	48.62
rs7823055	G	T	0.3507	0.0286	1.64E-34	150.11
rs9852014	G	A	0.6206	0.0493	2.26E-36	158.63
rs9274357	T	C	0.4574	0.0352	1.28E-38	168.91
rs17849501	T	C	0.8109	0.0499	1.81E-59	264.48

Table S4. 46 SNPs used as instrumental variables for RA in MR analysis

SNP	A1	A2	β	SE	*P*-value	*F*-statistics
rs10173253	A	G	0.0783	0.0138	1.32E-08	32.19
rs10905284	A	C	-0.0984	0.0149	3.81E-11	43.61
rs10972201	A	G	0.0994	0.0137	4.28E-13	52.64
rs10985070	A	C	-0.0786	0.0131	1.92E-09	36.00
rs113532504	T	C	0.1215	0.0215	1.61E-08	31.94
rs115284761	C	T	-0.1166	0.0210	2.78E-08	30.83
rs116548543	G	T	-0.5500	0.0576	1.30E-21	91.18
rs11810143	G	A	0.1148	0.0195	3.63E-09	34.66
rs12137270	T	C	0.0846	0.0151	2.20E-08	31.39
rs12474386	A	G	-0.1000	0.0129	1.03E-14	60.09
rs12506688	T	C	0.1341	0.0139	3.71E-22	93.07
rs12663951	T	C	-0.6656	0.0581	2.10E-30	131.24
rs137687	A	G	-0.0893	0.0130	7.79E-12	47.19
rs141060638	A	G	0.3739	0.0455	2.12E-16	67.53
rs143384650	C	T	-0.5660	0.0535	3.47E-26	111.92
rs16903108	C	T	-0.1420	0.0212	1.96E-11	44.86
rs1696466	C	T	0.0707	0.0129	3.94E-08	30.04
rs2045793	G	A	-0.0971	0.0153	2.37E-10	40.28
rs2240336	T	C	-0.0856	0.0134	1.89E-10	40.81
rs2561477	A	G	-0.0915	0.0139	4.96E-11	43.33
rs2664035	A	G	0.0815	0.0130	3.85E-10	39.30
rs2793108	T	C	0.0703	0.0129	4.62E-08	29.70
rs34673422	C	A	0.7845	0.0465	8.44E-64	284.63
rs41399051	C	T	-0.7191	0.0578	1.54E-35	154.78
rs4239702	C	T	0.1036	0.0144	5.98E-13	51.76
rs4584833	T	C	0.0840	0.0134	3.51E-10	39.30
rs4853458	G	A	-0.1236	0.0149	9.84E-17	68.81
rs4916340	T	G	0.0836	0.0148	1.52E-08	31.91
rs4938573	T	C	0.1221	0.0165	1.39E-13	54.76
rs548234	T	C	-0.0770	0.0133	7.30E-09	33.52
rs5745271	G	T	0.0749	0.0131	1.14E-08	32.69
rs61550563	C	A	-0.7602	0.0424	7.10E-72	321.46
rs6429207	C	A	-0.1009	0.0170	2.76E-09	35.23
rs6570194	C	A	0.1369	0.0235	5.53E-09	33.94
rs678347	A	G	-0.0835	0.0141	3.25E-09	35.07

rs706778	T	C	0.1048	0.0128	3.37E-16	67.04
rs7170151	T	C	0.1022	0.0143	9.49E-13	51.08
rs72928038	A	G	0.1068	0.0184	6.28E-09	33.69
rs7731626	A	G	-0.1860	0.0155	4.20E-33	144.00
rs8026898	A	G	0.1375	0.0140	9.90E-23	96.46
rs8133843	A	G	0.0851	0.0140	1.33E-09	36.95
rs9603608	C	A	-0.1087	0.0137	2.32E-15	62.95
rs998731	T	C	0.0771	0.0137	2.00E-08	31.67
rs114435492	C	T	-0.5142	0.0660	6.65E-15	60.70
rs17534670	A	G	-0.0904	0.0127	9.80E-13	50.67
rs3087243	A	G	-0.1208	0.0128	2.72E-21	89.07

Table S5. 32 SNPs used as instrumental variables for psoriasis in MR analysis

SNP	A1	A2	β	SE	*P*-value	*F*-statistics
rs10816608	T	G	-0.1026	0.0167	8.47E-10	37.75
rs11205044	T	C	-0.1866	0.0172	1.42E-27	117.70
rs11249215	A	G	0.1403	0.0160	2.14E-18	76.89
rs115059666	A	G	0.5147	0.0744	4.47E-12	47.86
rs11767350	A	G	0.0998	0.0164	1.19E-09	37.03
rs11795343	T	C	0.1062	0.0164	1.03E-10	41.93
rs12133684	A	G	0.1218	0.0206	3.26E-09	34.96
rs1295685	A	G	-0.1759	0.0206	1.16E-17	72.91
rs1648153	A	G	-0.1388	0.0164	2.93E-17	71.63
rs2301368	A	G	0.1073	0.0168	1.61E-10	40.79
rs2675662	A	G	0.1184	0.0167	1.49E-12	50.27
rs28998802	A	G	0.2113	0.0222	1.89E-21	90.59
rs39841	A	G	-0.1624	0.0176	2.56E-20	85.14
rs438650	T	C	-0.1244	0.0195	1.76E-10	40.70
rs565272	A	G	-0.1009	0.0182	3.06E-08	30.74
rs59960858	A	C	-0.2086	0.0243	1.06E-17	73.69
rs6063454	T	G	-0.1551	0.0166	1.08E-20	87.30
rs6894840	T	G	-0.1152	0.0166	3.42E-12	48.16
rs7141014	T	C	0.1123	0.0203	3.08E-08	30.60
rs73727477	A	G	-0.4866	0.0682	9.61E-13	50.91
rs73986523	T	C	0.2122	0.0380	2.41E-08	31.18
rs771576	T	C	-0.1029	0.0169	1.08E-09	37.07
rs8016947	T	G	-0.1476	0.0162	8.18E-20	83.01
rs9259397	T	C	0.2967	0.0221	3.87E-41	180.24
rs9504361	A	G	0.1068	0.0164	6.44E-11	42.41
rs9513593	A	G	-0.1197	0.0205	5.34E-09	34.09
rs9591325	T	C	0.1900	0.0324	4.50E-09	34.39
rs62396278	T	C	0.4702	0.0378	1.61E-35	154.73
rs12215963	A	G	-1.0578	0.0392	2.50E-160	728.17
rs10893885	A	G	0.1060	0.0161	3.97E-11	43.35

rs582757	T	C	-0.1846	0.0176	1.15E-25	110.01
rs9481169	T	G	0.3716	0.0263	2.47E-45	199.64

Table S6. 41 SNPs used as instrumental variables for MS in MR analysis

SNP	A1	A2	β	SE	*P*-value	*F*-statistics
rs10063294	A	G	-0.0990	0.0163	1.13E-09	37.09
rs1014486	C	T	0.1051	0.0164	1.36E-10	41.21
rs10801908	C	T	0.2150	0.0264	3.54E-16	66.48
rs11256593	T	C	0.1863	0.0174	6.78E-27	115.29
rs12622670	T	C	0.1068	0.0165	1.04E-10	41.74
rs12925972	C	T	0.0946	0.0171	3.07E-08	30.66
rs1465697	C	T	-0.1243	0.0188	3.48E-11	43.89
rs17124032	G	A	0.2168	0.0316	7.08E-12	47.00
rs1738074	T	C	-0.1137	0.0167	9.91E-12	46.35
rs2248461	A	G	-0.1081	0.0174	5.33E-10	38.55
rs2317231	G	T	0.1006	0.0167	1.90E-09	36.07
rs2546890	A	G	0.1170	0.0164	1.04E-12	50.77
rs2681424	T	C	0.1212	0.0166	2.71E-13	53.41
rs28703878	A	G	-0.1336	0.0214	4.51E-10	38.88
rs34695601	T	C	0.1095	0.0198	3.17E-08	30.60
rs354033	G	A	0.1080	0.0189	1.21E-08	32.48
rs35486093	A	G	-0.1795	0.0281	1.60E-10	40.90
rs35703946	G	A	0.1725	0.0287	1.94E-09	36.04
rs438613	C	T	0.1380	0.0166	9.43E-17	69.08
rs478093	A	G	-0.1051	0.0179	4.30E-09	34.48
rs55858457	G	T	-0.1131	0.0198	1.20E-08	32.49
rs56232455	G	A	-0.1584	0.0281	1.78E-08	31.72
rs6032662	C	T	0.1338	0.0183	2.85E-13	53.31
rs62420820	A	G	0.1372	0.0188	2.50E-13	53.57
rs6496663	A	C	-0.1006	0.0181	2.78E-08	30.86
rs6670198	C	T	-0.1450	0.0176	2.03E-16	67.57
rs6763437	G	A	0.7791	0.1321	3.72E-09	34.76
rs6990534	A	G	-0.1071	0.0182	3.60E-09	34.83
rs701006	G	A	0.1139	0.0168	1.35E-11	45.74
rs71542422	T	C	-1.1264	0.0492	3.43E-116	525.02
rs7190580	A	G	0.0980	0.0179	4.64E-08	29.86
rs72928038	G	A	-0.1605	0.0248	9.01E-11	42.03
rs9259366	C	T	-0.3246	0.0242	3.86E-41	180.45
rs9591325	T	C	0.2124	0.0340	4.16E-10	39.04
rs9610458	T	C	0.1142	0.0165	4.57E-12	47.86
rs9955954	G	A	-0.1100	0.0195	1.54E-08	32.00
rs9992763	T	G	-0.0900	0.0165	4.51E-08	29.92
rs1077667	C	T	0.1519	0.0212	8.37E-13	51.19
rs4325907	T	C	-0.0993	0.0168	3.68E-09	34.78

rs57116599	A	G	-0.1200	0.0202	2.59E-09	35.47
rs12365699	G	A	0.1438	0.0228	3.15E-10	39.58

Table S7. 7 SNPs used as instrumental variables for JIA in MR analysis

SNP	A1	A2	β	SE	*P*-value	*F*-statistics
rs11889341	T	C	0.2202	0.0345	1.83E-10	40.64
rs144118870	G	A	0.9713	0.0882	3.50E-28	121.17
rs3025650	C	T	-0.3896	0.0657	3.01E-09	35.18
rs497523	C	T	-0.2004	0.0346	7.12E-09	33.50
rs6679677	A	C	0.3628	0.0487	9.18E-14	55.53
rs7731626	A	G	-0.2543	0.0345	1.76E-13	54.26
rs9960807	G	A	0.2611	0.0433	1.58E-09	36.44

Table S8. 17 SNPs used as instrumental variables for CD in MR analysis

SNP	A1	A2	β	SE	*P*-value	*F*-statistics
rs1050976	T	C	-0.1110	0.0185	1.84E-09	36.14
rs11753629	A	G	-0.3313	0.0302	4.71E-28	120.59
rs11851414	C	T	0.1204	0.0221	4.71E-08	29.83
rs12068671	C	T	-0.1567	0.0244	1.40E-10	41.17
rs1378938	A	G	0.1178	0.0204	7.79E-09	33.33
rs1893592	C	A	-0.1239	0.0209	2.96E-09	35.21
rs2030519	G	A	-0.2783	0.0189	3.00E-49	217.61
rs2097282	C	T	0.1840	0.0197	1.13E-20	86.92
rs55743914	T	C	0.1873	0.0212	1.15E-18	77.79
rs61579022	A	G	0.1080	0.0188	9.92E-09	32.86
rs6498114	G	T	0.1310	0.0212	5.83E-10	38.38
rs6715106	G	A	-0.2374	0.0412	8.38E-09	33.19
rs76830965	A	C	0.3075	0.0284	2.57E-27	117.22
rs13198474	A	G	0.9462	0.0312	1.00E-200	920.79
rs1018326	C	T	0.1519	0.0186	3.06E-16	66.76
rs10790269	C	T	-0.1572	0.0240	5.44E-11	43.01
rs79758729	G	A	0.1630	0.0291	2.12E-08	31.39

Table S9. 11 SNPs used as instrumental variables for PSC in MR analysis

SNP	A1	A2	β	SE	*P*-value	*F*-statistics
rs114581973	C	T	0.5342	0.1020	3.40E-08	27.42
rs139010734	T	C	3.3554	0.1400	1.98E-154	574.41
rs145832854	G	A	0.6243	0.1190	2.58E-08	27.53
rs231389	C	T	0.2062	0.0360	4.42E-09	32.81
rs34645399	G	A	0.7467	0.0470	1.64E-59	252.40
rs4147359	A	G	0.2167	0.0300	4.06E-13	52.19
rs4817988	G	A	0.3148	0.0410	4.20E-15	58.96
rs79940565	C	T	0.7631	0.1460	2.00E-08	27.32
rs80060485	C	T	0.3457	0.0620	8.54E-09	31.09

rs41316239	G	A	0.5682	0.0900	4.97E-11	39.85
rs72837826	T	G	0.3038	0.0510	1.26E-09	35.48

Table S10. The results of MR analysis between PSC and the CRC validation cohort.

	MR method	OR (95% CI)	*P*	Pleiotropy test *P*	Heterogeneity test *P*
PSC on CRC (validation)	IVW	1.0005 (1.0001-1.0010)	0.025	0.532	0.426
	MR-Egger	1.0006 (1.0001-1.0012)	0.043		
	WM	1.0007 (1.0001-1.0012)	0.017		

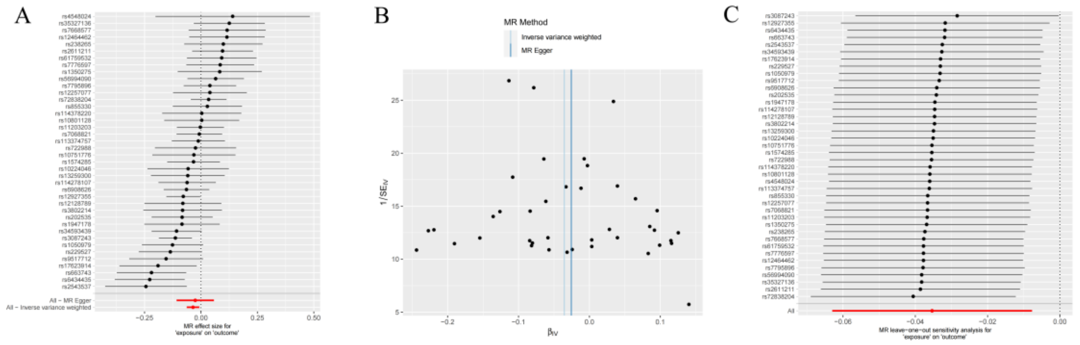

Figure S1. (A) Forest plot of T1D on CRC for MR analysis. (B) Funnel plot of T1D on CRC for MR analysis. (C) Leave-one-out sensitivity analysis plot of T1D on CRC for MR analysis.

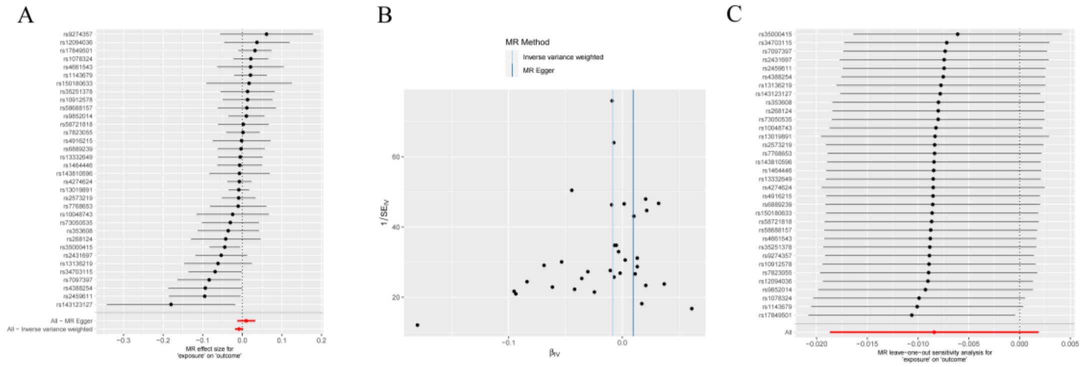

Figure S2. (A) Forest plot of SLE on CRC for MR analysis. (B) Funnel plot of SLE on CRC for MR analysis. (C) Leave-one-out sensitivity analysis plot of SLE on CRC for MR analysis.

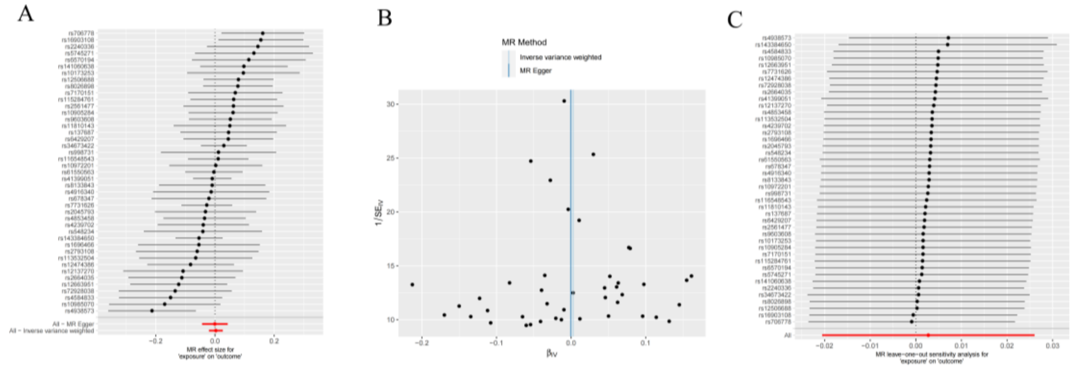

Figure S3. (A) Forest plot of RA on CRC for MR analysis. (B) Funnel plot of RA on CRC for MR analysis. (C) Leave-one-out sensitivity analysis plot of RA on CRC for MR analysis.

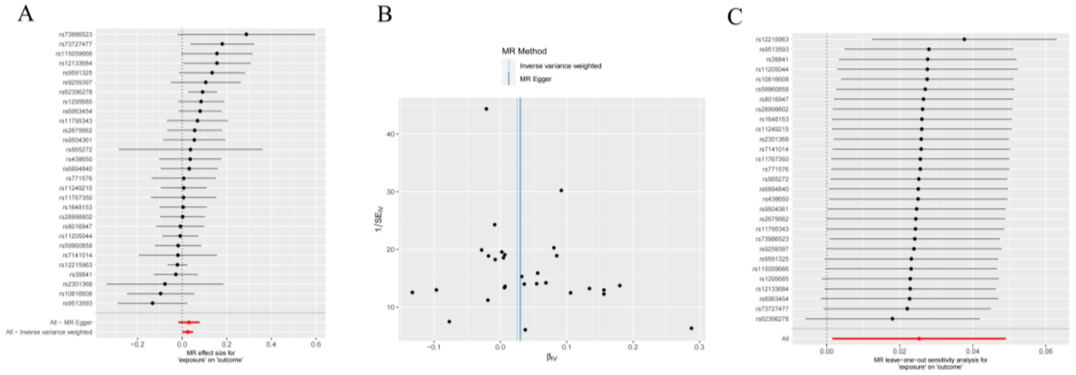

Figure S4. (A) Forest plot of psoriasis on CRC for MR analysis. (B) Funnel plot of psoriasis on CRC for MR analysis. (C) Leave-one-out sensitivity analysis plot of psoriasis on CRC for MR analysis.

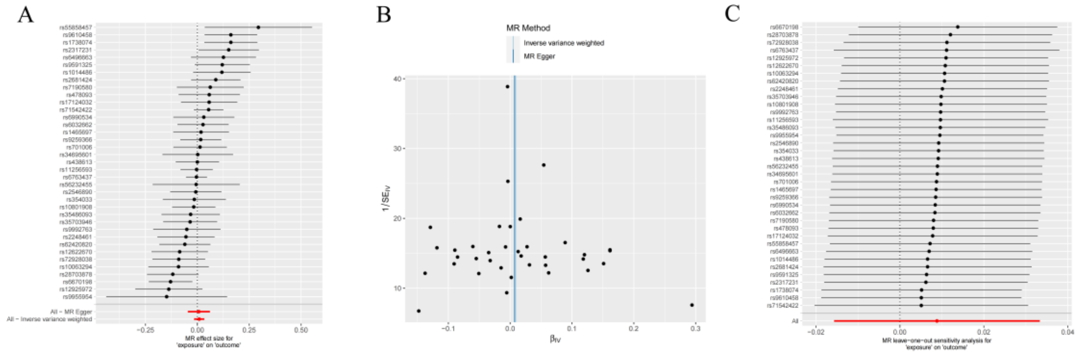

Figure S5. (A) Forest plot of MS on CRC for MR analysis. (B) Funnel plot of MS on CRC for MR analysis. (C) Leave-one-out sensitivity analysis plot of MS on CRC for MR analysis.

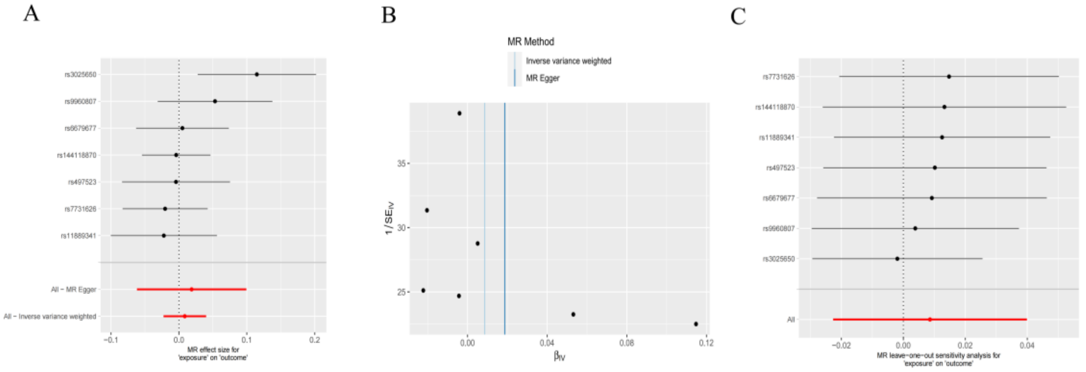

Figure S6. (A) Forest plot of JIA on CRC for MR analysis. (B) Funnel plot of JIA on CRC for MR analysis. (C) Leave-one-out sensitivity analysis plot of JIA on CRC for MR analysis.

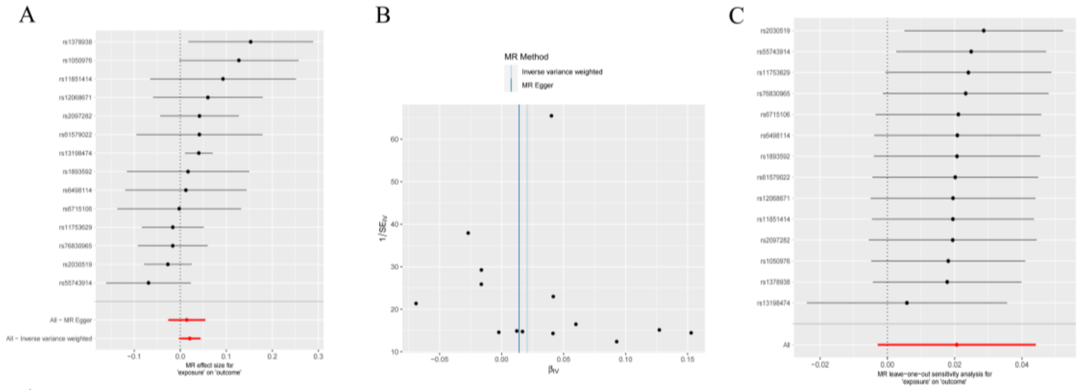

Figure S7. (A) Forest plot of CD on CRC for MR analysis. (B) Funnel plot of CD on CRC for MR analysis. (C) Leave-one-out sensitivity analysis plot of CD on CRC for MR analysis.

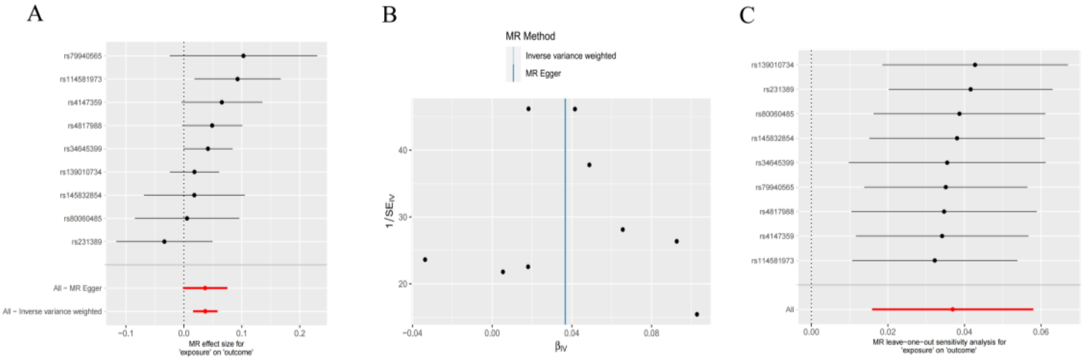

Figure S8. (A) Forest plot of PSC on CRC for MR analysis. (B) Funnel plot of PSC on CRC for MR analysis. (C) Leave-one-out sensitivity analysis plot of PSC on CRC for MR analysis.

Table S11. The details of instrumental variables for CRC.

SNP	A1	A2	β	SE	*P*-value	*F*-statistics
rs10159108	A	G	0.0663	0.0120	2.83E-08	30.82
rs12751610	C	T	-0.0588	0.0098	2.37E-09	35.65
rs61776719	A	C	-0.0582	0.0076	2.50E-14	58.10
rs12143541	G	A	0.0825	0.0102	7.99E-16	64.87
rs2651244	A	G	-0.0441	0.0075	4.31E-09	34.48
rs4916282	C	T	-0.0406	0.0074	4.56E-08	29.90
rs8179460	C	T	-0.0771	0.0074	3.51E-25	107.47
rs12078075	A	G	-0.0714	0.0127	1.94E-08	31.55
rs12140529	T	C	0.0837	0.0090	1.01E-20	87.13
rs11255841	A	T	-0.0987	0.0080	8.44E-35	151.43
rs10821905	A	G	-0.0670	0.0096	3.58E-12	48.34
rs704017	G	A	0.0866	0.0076	5.29E-30	129.49
rs1250568	C	T	0.0486	0.0075	1.04E-10	41.75
rs2193352	G	A	0.0949	0.0093	2.74E-24	103.40
rs56188434	A	G	0.1022	0.0125	3.46E-16	66.52
rs2296782	G	A	0.0555	0.0086	1.25E-10	41.38
rs10160763	T	G	-0.0415	0.0075	2.61E-08	30.98

rs174583	T	C	-0.0540	0.0078	4.11E-12	48.07
rs10899024	G	A	0.0708	0.0075	5.65E-21	88.29
rs117042741	T	C	-0.2261	0.0197	1.43E-30	132.08
rs2155065	T	C	-0.0408	0.0074	2.83E-08	30.82
rs7130173	C	A	-0.1267	0.0081	1.64E-55	246.33
rs3217810	T	C	0.1232	0.0116	1.96E-26	113.19
rs10849438	G	T	0.0807	0.0113	1.05E-12	50.75
rs11169572	C	T	0.0860	0.0075	2.72E-30	130.81
rs7398375	G	C	-0.0639	0.0088	3.15E-13	53.11
rs11178634	T	G	-0.0456	0.0075	1.23E-09	36.92
rs10859923	C	T	0.0509	0.0076	1.96E-11	45.02
rs653178	T	C	0.0757	0.0074	2.29E-24	103.75
rs6490020	C	T	0.0642	0.0077	7.57E-17	69.52
rs12427846	C	T	0.0791	0.0088	3.06E-19	80.40
rs45597035	G	A	-0.0614	0.0077	2.02E-15	63.05
rs1924816	G	A	-0.0590	0.0089	2.66E-11	44.41
rs4773184	T	C	0.0578	0.0077	4.97E-14	56.74
rs35107139	C	A	0.0893	0.0084	2.87E-26	112.43
rs12879025	A	G	-0.0562	0.0082	5.59E-12	47.47
rs8020436	A	G	0.0604	0.0082	1.48E-13	54.59
rs2293582	A	G	0.1479	0.0091	1.10E-59	265.47
rs72717673	T	C	-0.0533	0.0095	1.75E-08	31.75
rs144674978	T	C	0.2933	0.0332	9.66E-19	78.13
rs56324967	C	T	0.0553	0.0080	4.62E-12	47.84
rs8031386	A	C	0.0476	0.0084	1.80E-08	31.70
rs12913420	C	G	0.0474	0.0084	1.58E-08	31.95
rs7179095	A	G	0.0625	0.0098	2.10E-10	40.37
rs9924886	C	A	-0.0559	0.0083	2.20E-11	44.79
rs12930889	T	C	-0.0599	0.0074	8.60E-16	64.73
rs12447408	A	G	0.0579	0.0086	1.81E-11	45.16
rs7199483	T	C	0.0616	0.0092	2.15E-11	44.83
rs73975588	C	A	-0.0891	0.0110	6.62E-16	65.24
rs1078643	A	G	0.0763	0.0092	9.32E-17	69.11
rs983318	A	G	0.0578	0.0085	1.06E-11	46.22
rs35204860	C	T	0.0775	0.0088	1.16E-18	77.77
rs7226855	G	A	-0.1681	0.0074	2.55E-114	516.42
rs10409772	A	C	0.0771	0.0138	2.11E-08	31.39
rs34797592	T	C	0.0900	0.0117	1.19E-14	59.55
rs28840750	G	T	-0.1912	0.0172	8.90E-29	123.89
rs11670143	G	A	0.0520	0.0076	8.19E-12	46.72
rs11670192	A	G	0.0600	0.0094	1.80E-10	40.67
rs11692435	A	G	-0.0910	0.0133	7.16E-12	46.98
rs7422195	A	G	0.0444	0.0076	4.93E-09	34.22
rs34119476	G	A	0.0483	0.0075	1.02E-10	41.78

rs4675253	G	C	0.0631	0.0079	1.06E-15	64.31
rs3731861	C	T	-0.0642	0.0077	5.28E-17	70.23
rs966816	A	G	-0.0979	0.0078	3.40E-36	157.81
rs6140071	T	C	0.0760	0.0077	1.09E-22	96.10
rs6140404	T	G	0.0863	0.0103	7.21E-17	69.62
rs2179593	A	C	0.0579	0.0083	2.57E-12	48.99
rs6066825	G	A	-0.0756	0.0077	1.42E-22	95.58
rs6020486	G	A	0.0695	0.0077	1.50E-19	81.81
rs6014965	G	A	-0.0466	0.0074	2.89E-10	39.75
rs1741640	C	T	0.1280	0.0090	7.71E-46	201.98
rs2839223	A	G	-0.0661	0.0110	1.63E-09	36.38
rs17003993	C	T	0.0595	0.0108	3.56E-08	30.38
rs12484832	T	A	-0.0413	0.0073	1.84E-08	31.65
rs5751474	A	G	0.0519	0.0090	8.97E-09	33.05
rs3747239	C	A	0.0488	0.0078	4.03E-10	39.10
rs35470271	G	A	0.0936	0.0102	4.09E-20	84.38
rs2001732	T	C	-0.0759	0.0107	1.47E-12	50.09
rs6781752	A	G	0.0510	0.0092	2.97E-08	30.73
rs13086367	G	A	-0.0527	0.0074	9.66E-13	50.91
rs72942485	A	G	-0.1785	0.0276	9.54E-11	41.91
rs4854776	A	C	-0.0423	0.0074	1.03E-08	32.79
rs113569514	C	T	-0.0666	0.0107	5.36E-10	38.54
rs35446936	A	G	-0.0534	0.0086	6.23E-10	38.25
rs280097	C	T	0.0520	0.0075	4.93E-12	47.72
rs7679673	A	C	-0.0583	0.0076	2.14E-14	58.40
rs7688025	A	G	-0.0588	0.0105	2.19E-08	31.32
rs11727676	C	T	0.0927	0.0125	1.02E-13	55.32
rs2735940	G	A	0.0863	0.0074	3.68E-31	134.79
rs1445012	C	G	0.0942	0.0081	1.67E-31	136.35
rs4976270	T	C	-0.0589	0.0074	2.02E-15	63.05
rs2302275	G	C	0.0479	0.0075	2.14E-10	40.33
rs41302867	A	G	-0.0698	0.0116	1.68E-09	36.32
rs2070699	T	G	0.0541	0.0074	3.75E-13	52.77
rs17197658	G	A	-0.0532	0.0087	9.93E-10	37.34
rs9271770	A	G	0.0839	0.0098	9.35E-18	73.64
rs4267954	A	G	0.0456	0.0079	7.37E-09	33.43
rs9470361	A	G	0.0653	0.0085	1.35E-14	59.31
rs6933790	C	T	-0.0798	0.0100	1.81E-15	63.26
rs62404966	T	C	-0.0656	0.0086	2.86E-14	57.83
rs72872212	A	G	0.1272	0.0205	5.12E-10	38.63
rs145997965	C	T	0.1895	0.0333	1.26E-08	32.39
rs6911915	C	T	0.0440	0.0074	2.71E-09	35.38
rs151127921	T	C	0.1593	0.0288	3.19E-08	30.59
rs3735491	A	C	-0.0488	0.0084	5.41E-09	34.04

rs4720558	T	C	-0.0448	0.0075	2.26E-09	35.73
rs6948177	G	A	0.0609	0.0078	8.61E-15	60.19
rs7806956	A	T	-0.0486	0.0084	6.15E-09	33.79
rs2527927	A	G	-0.0419	0.0073	1.22E-08	32.46
rs17686932	G	A	-0.1126	0.0167	1.59E-11	45.42
rs2437841	A	G	-0.0561	0.0094	2.42E-09	35.60
rs16892766	C	A	0.2010	0.0128	6.10E-56	248.30
rs11775667	A	G	0.0561	0.0094	2.24E-09	35.75
rs6983267	T	G	-0.1510	0.0074	1.46E-93	421.03
rs1537372	T	G	-0.0632	0.0075	2.75E-17	71.52
rs10978941	T	C	-0.0575	0.0092	3.79E-10	39.22

Table S12. Pleiotropy and heterogeneity test of the reverse MR analysis.

Outcome	Pleiotropy test			Heterogeneity test		
	MR-Egger			Inverse variance weighted		
	Intercept	SE	*P*	Q	Q_df	Q_*p*val
T1D	0.007	0.006	0.305	214.512	103	< 0.001
Psoriasis	-0.002	0.007	0.783	186.241	103	< 0.001
PSC	0.010	0.013	0.443	81.205	8	0.061

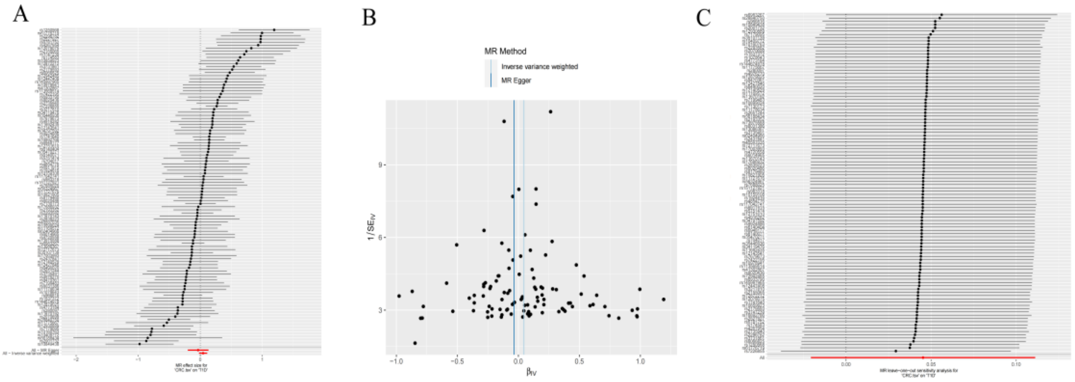

Figure S9. (A) Forest plot of CRC on T1D for reverse MR analysis. (B) Funnel plot of CRC on T1D for reverse MR analysis. (C) Leave-one-out sensitivity analysis plot of CRC on T1D for reverse MR analysis.

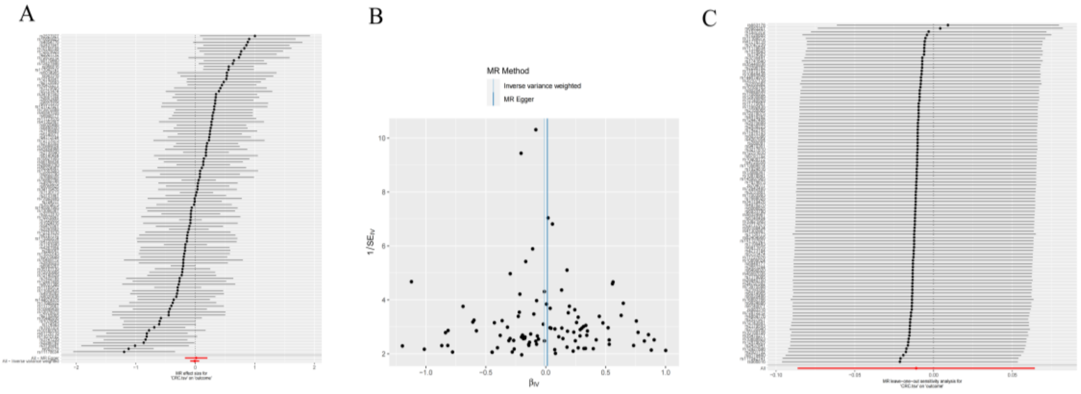

Figure S10. (A) Forest plot of CRC on psoriasis for reverse MR analysis. (B) Funnel plot of CRC on

psoriasis for reverse MR analysis. (C) Leave-one-out sensitivity analysis plot of CRC on psoriasis for reverse MR analysis.

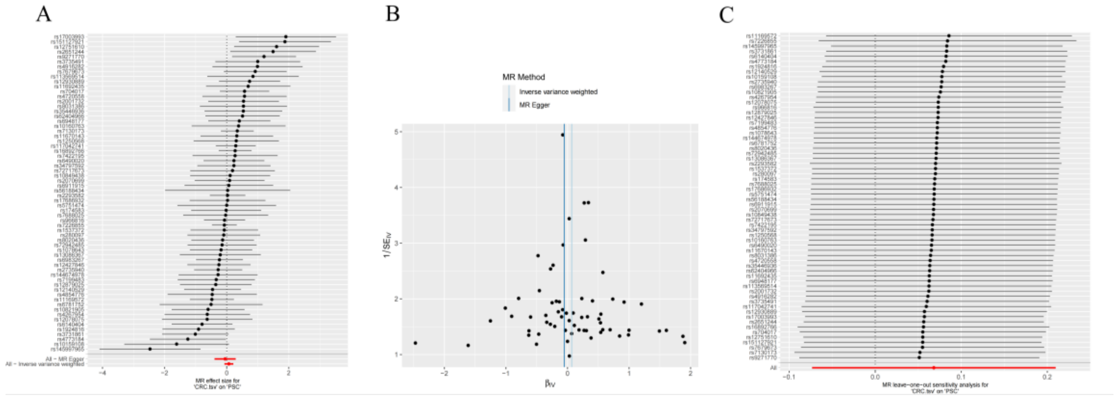

Figure S11. (A) Forest plot of CRC on PSC for reverse MR analysis. (B) Funnel plot of CRC on PSC for reverse MR analysis. (C) Leave-one-out sensitivity analysis plot of CRC on PSC for reverse MR analysis.
